# 981. Sink drain environmental hygiene intervention successfully reduces burden of AROs

**DOI:** 10.1093/ofid/ofad500.036

**Published:** 2023-11-27

**Authors:** Erin Newcomer, Carrie O’Neal, Lucy Vogt, David Macdonald, Candice Cass, Meghan Wallace, Tiffany Hink, Francesca Yerbic, Carol Muenks, Rebecca Gordon, Henry B Stewart, Mostafa Amor, Kevin Jolani, Kelly Alvarado, Alyssa Valencia, Carleigh Samuels, Kate Peacock, Daniel Park, Emily Struttman, Kimberley Sukhum, Carey-Ann Burnham, Gautam Dantas, Jennie H Kwon

**Affiliations:** Washington University in St. Louis, St. Louis, MO; Washington University in St. Louis, St. Louis, MO; Washington University, St. Louis, Missouri; Washington University in St. Louis, St. Louis, MO; Washington University, St. Louis, Missouri; Washington university School of Medicine, Hillsboro, Missouri; Washington University, St. Louis, Missouri; Washington University in St. Louis, St. Louis, MO; Washington University School of Medicine, Saint Louis, MO; Washington University in St. Louis, St. Louis, MO; Washington University School of Medicine in St. Louis, Indianapolis, Indiana; Washington University in St. Louis, St. Louis, MO; Washington University in St. Louis, St. Louis, MO; Washington University in St. Louis, St. Louis, MO; Washington University in St. Louis, St. Louis, MO; Washington University in St. Louis, St. Louis, MO; Washington University in St. Louis, St. Louis, MO; Washington University School of Medicine, Saint Louis, MO; Washington University in St. Louis, St. Louis, MO; Washington University in St. Louis, St. Louis, MO; Pattern Bioscience , Saint Louis, MO; Washington University School of Medicine in St Louis, St. Louis, MO; Washington University - School of Medicine, St. Louis, MO

## Abstract

**Background:**

Healthcare associated infections (HAIs) and outbreaks due to hospital sinks have been noted for many years, however, there remains no standardized environmental hygiene protocol to combat these HAIs. The goal of this study was to evaluate the impact of a sink environmental hygiene intervention on gram negative microbial load.

**Methods:**

We implemented our intervention across 18 rooms in two ICUs at Barnes Jewish Hospital in St. Louis, USA (Figure 1). At low frequency (1x/week) and high frequency (5x/week) intervals, we wiped sink surfaces with 10% bleach wipes and pumped (Foam-It Pump, FOAMit, Grand Rapids, MI) hydrogen-peroxide based foam (Virasept, Ecolab, St. Paul, MN) into sink drains, allowing a contact time of 3 minutes before rinsing. Control rooms received no intervention. We used E-swabs (Copan, Murrieta, CA) to swab sink drains and surrounding surfaces during a pre-intervention, two intervention, and two post-intervention periods. Swabs and water samples were selectively cultured for AROs, and isolates were identified using MALDI-TOF MS. We also obtained information on HAIs that occurred in the ICUs during the study period.

Study design overview.

**Figure:**
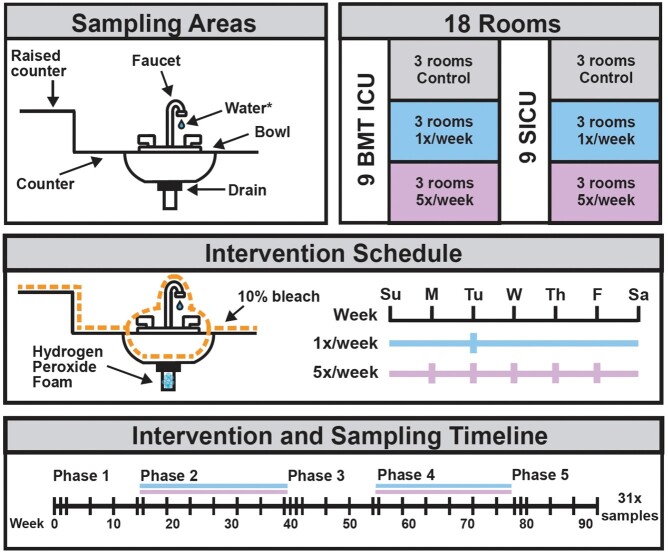

We tested 2 frequencies of a disinfectant foam pump and bleach wipe intervention in 18 rooms across a SCTO ICU and SICU at Barnes Jewish Hospital. We sampled multiple surfaces including sink drains across a baseline phase (Phase 1), intervention phase (Phase 2), post-intervention phase (Phase 3), a second intervention phase (Phase 4), and another post intervention phase (Phase 5).

**Results:**

Sink drains yielded the most unique AROs compared to other surfaces. The most identified AROs were *P. aeruginosa* and *S. maltophilia* (Figure 2). The intervention reduced total microbial and gram-negative burden at both frequencies, with the high frequency reducing the fraction of sink drains yielding gram-negatives by >80% (p< 0.05, Figure 3). The low and high frequencies reduced the fraction of sink drains yielding *P. aeruginosa* (62-82% and 81-100%, respectively) or *S. maltophilia* (44-68% and 72-94%, respectively); for many weeks neither was recovered (Figure 4). The number of HAIs (n=69) recorded was insufficient to comment on impacts on HAIs.

In-room sink drains yielded the most AROs.

**Figure:**
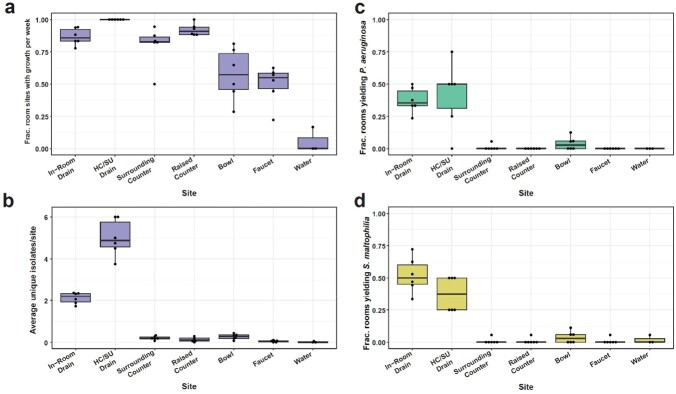

a) Average weekly fraction of rooms with growth on BAP from each site. Average weekly b) unique ARO isolates c) fraction of rooms yielding P. aeruginosa and d) fraction of rooms yielding S. maltophilia. Permutation test, BH corrected.

Both interventions reduced the fraction of rooms with culturable growth.

**Figure:**
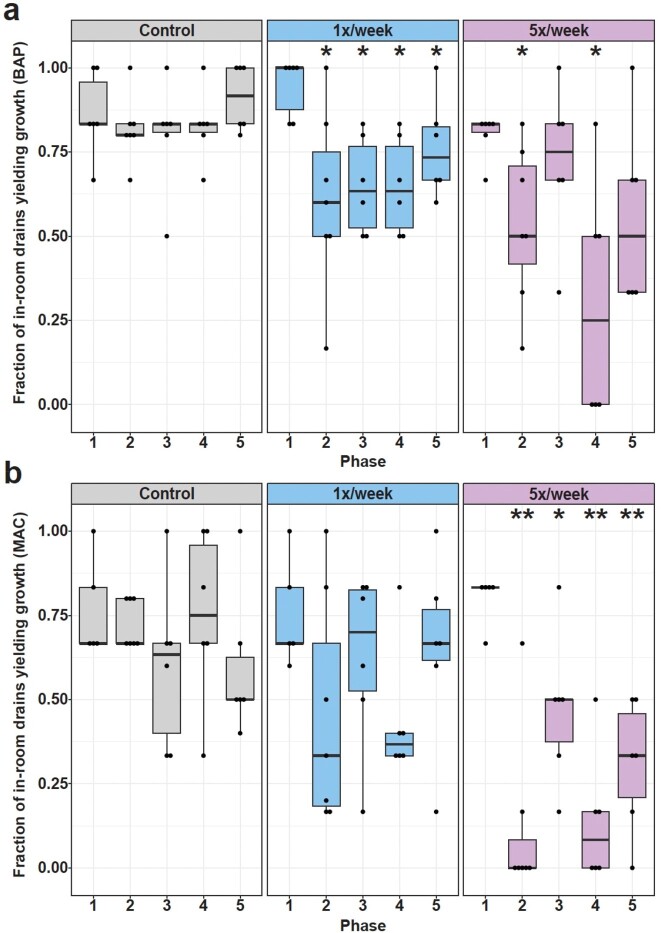

Average weekly fraction of rooms in each intervention group that yielded culturable growth on a) BAP and b) MAC plates. Dunn’s test with BH correction was used to evaluate differences between groups in Phase 1 (baseline). Changes within groups versus Phase 1 were tested using permutation tests and BH corrected. Significant changes are denoted above the phase that has changed versus Phase 1 as follows: *: p ≤ 0.05, **: p ≤ 0.01.

Interventions reduced the fraction of in-room sink drains yielding ARO isolates.

**Figure:**
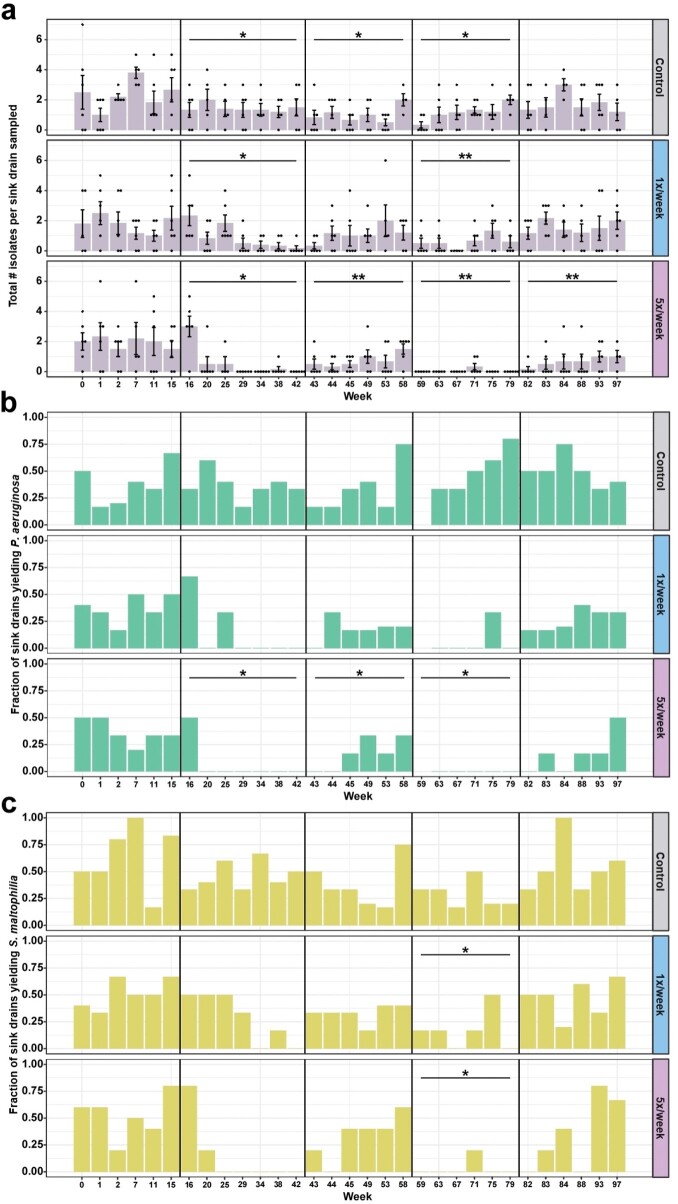

a) Weekly counts per sink drain of unique AROs. Fraction of rooms yielding b) P. aeruginosa and c) S. maltophilia from in-room sink drain. Both interventions significantly reduced culturable ARO isolates, and frequently resulted in no P. aeruginosa or S. maltophilia collected for multiple weeks. Significant changes are denoted above the phase that has changed versus Phase 1 as follows: *: p ≤ 0.05, **: p ≤ 0.01.

**Conclusion:**

As antibiotic resistance continues to rise, preventing HAIs caused by AROs becomes increasingly important. There are no standardized sink drain environmental hygiene protocols, even as outbreaks tied to sink drain colonization are regularly reported. This protocol effectively reduced ARO colonization of sinks drains. While larger studies are needed to observe effects of this intervention on HAIs, this study provides a promising tool for combatting sink drain-associated HAIs.

**Disclosures:**

**Carey-Ann Burnham, PhD**, Pattern Bioscience: Stocks/Bonds

